# Coffee and Physical Performance: What Is Driven by Caffeine and What Is Not

**DOI:** 10.3390/nu18162690

**Published:** 2026-08-18

**Authors:** Joanna Grzelczyk, Joanna Ziętala, Grażyna Budryn, Mariusz Konieczny, Przemysław Domaszewski

**Affiliations:** 1 Institute of Food Technology and Analysis, Faculty of Biotechnology and Food Sciences, Lodz University of Technology, 90-537 Lodz, Poland; joanna.grzelczyk@p.lodz.pl (J.G.); joanna.zietala@dokt.p.lodz.pl (J.Z.); grazyna.budryn@p.lodz.pl (G.B.); 2 Faculty of Physical Education and Physiotherapy, Opole University of Technology, 45-758 Opole, Poland; m.konieczny@po.edu.pl; 3 Institute of Health Sciences, University of Opole, Katowicka 68, 45-060 Opole, Poland

**Keywords:** ergogenic aids, endurance exercise, perceived exertion, muscle pain, recovery processes, circadian rhythm

## Abstract

Coffee is widely consumed by physically active individuals and athletes, often with the intention of enhancing alertness, reducing fatigue and supporting exercise performance. Its performance-related effects are primarily attributed to caffeine; however, responses to coffee consumption vary substantially across individuals and type of training. This narrative review examines current evidence on the role of coffee consumption in sport, with particular emphasis on caffeine-related mechanisms, physical performance, fatigue, muscle pain and recovery, as well as factors modifying individual responses, including circadian rhythm, habitual intake and genetic variability. Available evidence indicates that coffee consumption prior to physical activity may support endurance performance, reduce perceived exertion and attenuate muscle pain, particularly during prolonged or demanding exercise and under conditions of sleep restriction or circadian misalignment. In contrast, effects on muscle strength and power performance appear less consistent and more context dependent. Interindividual differences in caffeine metabolism, sensitivity to stimulation and susceptibility to sleep disruption further influence both the effectiveness and tolerability of coffee intake. Emerging findings also suggest that coffee, when consumed in combination with carbohydrates, may enhance post-exercise muscle glycogen resynthesis, a potential benefit of particular relevance for endurance athletes facing limited recovery time. At the same time, evidence regarding non-caffeine bioactive compounds present in coffee remains limited and largely indirect, indicating a modulatory rather than ergogenic role. Overall, coffee should not be regarded as a universally effective ergogenic aid, as its effects may vary according to individual characteristics, training demands and the timing of consumption. Its effective use requires careful consideration of individual characteristics, training demands and timing of consumption, underscoring the importance of personalized strategies in both performance and recovery settings.

## 1. Introduction

Coffee is among the most widely consumed beverages worldwide, valued primarily for its sensory qualities and its stimulating properties. In recent years, however, coffee has attracted increasing attention beyond its cultural and hedonic roles, particularly in the context of human health. A growing body of evidence indicates that habitual coffee consumption is associated with anti-inflammatory, antidiabetic and neuroprotective effects, as well as with a lower oxidative burden. Collectively, these observations have supported the hypothesis that regular coffee intake may contribute to a reduced risk of several chronic lifestyle-related diseases [1,2,3,4,5,6].

Beyond its relevance to general health, coffee has acquired distinct practical significance in the context of physical activity and sport. Among physically active individuals and athletes, coffee consumption is often intentional rather than incidental and frequently aligned with specific training sessions or competitive demands. Instead of being consumed solely as part of everyday dietary habits, coffee is commonly used with the aim of enhancing alertness, mitigating fatigue and supporting exercise performance, particularly in physically or cognitively demanding training and competition settings [7].

The performance-related effects of coffee have traditionally been attributed to its caffeine content (1,3,7-trimethylxanthine), a compound that has been extensively investigated within the field of sports nutrition [8]. Research conducted over several decades indicates that caffeine intake can support endurance performance and, to a lesser extent, strength and power output across a wide range of sport disciplines, in both recreationally active individuals and elite athletes [9,10,11,12]. Beneficial effects are most consistently reported at doses of approximately 3–6 mg per kilogram of body mass; however, considerable interindividual variability in response has been observed. This variability suggests that factors beyond total body mass, including body composition, may play an important role in determining the effective and tolerable dose of caffeine [13].

In addition to its effects on physical performance, caffeine influences multiple aspects of cognitive functioning, including vigilance, reaction time, mood and perceived exertion. These effects become particularly relevant under conditions of sleep restriction or mental fatigue, which are common in contemporary sport due to early training schedules, accumulated training load, congested competition calendars and travel across time zones [14]. In such contexts, improvements in alertness and attentional control may translate into practical advantages, such as more effective pacing strategies, improved decision-making during competition and greater consistency of technical execution, especially during prolonged or high-intensity exercise [15].

Importantly, responses to coffee consumption are far from uniform. Genetic background, habitual caffeine intake, circadian rhythm, timing of ingestion and training status all modulate both the magnitude and reliability of observed effects [16,17,18]. In addition, coffee is rarely consumed in isolation but rather as part of a broader dietary pattern and training routine, which may further shape its physiological impact during exercise and recovery. The caffeine content of coffee beverages is also highly variable and depends on factors such as coffee species, degree of roasting and brewing method. Consequently, generalized recommendations based on the number of cups consumed, for example, statements suggesting that the intake of up to four cups of coffee is universally safe in individuals without hypersensitivity, should be interpreted with caution. Such guidance does not adequately account for variability in caffeine content or individual response, and estimates based solely on cup number should therefore be regarded as approximate rather than precise.

Taken together, these considerations indicate that the effects of coffee consumption on physical performance cannot be assumed to be universal, but may vary according to individual characteristics, habitual caffeine intake, exercise demands and the timing of consumption. Moreover, although caffeine represents the most extensively studied and clearly defined component of coffee in relation to exercise performance, coffee is a complex biological matrix containing numerous additional bioactive compounds. Increasing interest has therefore emerged in the potential contribution of non-caffeine constituents of coffee to exercise-related outcomes, particularly through their influence on fatigue, recovery and metabolic regulation.

Accordingly, this narrative review examines the role of coffee consumption in the context of physical performance and training in physically active individuals. Particular emphasis is placed on caffeine-related mechanisms, individual variability in response, and the influence of circadian and genetic factors on exercise outcomes. In addition, available evidence concerning non-caffeine bioactive compounds present in coffee is discussed in relation to their potential modulatory role in physical activity and recovery. By integrating findings from exercise physiology, neuroscience and nutritional biochemistry, this review aims to clarify how and under what conditions coffee may function as a context-dependent element of sports nutrition, rather than as a universally effective ergogenic aid.

## 2. Methodology of the Review

The literature search covered studies published between 2000 and 2026 and was conducted using PubMed, Scopus and the Web of Science Core Collection. Search terms included coffee, caffeine, physical performance, endurance, muscle strength, fatigue, recovery, circadian rhythm, genetics, CYP1A2 and sports nutrition. Additional relevant publications were identified by screening the reference lists of selected articles. Studies were selected based on their relevance to the topics addressed in this review, with preference given to peer-reviewed original research, systematic reviews, meta-analyses and position statements. Evidence concerning caffeine was drawn primarily from human intervention studies evaluating exercise-related outcomes. For non-caffeine coffee constituents, mechanistic, metabolic and recovery-oriented studies were also considered because exercise-specific evidence remains limited. Given the narrative nature of this review, the literature was evaluated qualitatively, considering the consistency of findings, study design and relevance to the topics addressed. No predefined eligibility criteria, formal study selection procedure, protocol registration, risk-of-bias assessment or meta-analysis were applied.

## 3. Caffeine and Physical Activity

### 3.1. Bioavailability of Caffeine

Caffeine is a relatively hydrophobic compound, which facilitates its rapid absorption from the gastrointestinal tract and enables efficient passage across biological membranes, including the blood–brain barrier [19]. Following oral ingestion, caffeine appears in the bloodstream within minutes, with peak plasma concentrations typically reached between 30 and 120 min, depending on the individual and the form of ingestion [20,21].

Despite undergoing hepatic metabolism, caffeine exhibits very high oral bioavailability, approaching 100%, indicating that first-pass metabolism does not substantially limit its systemic availability [22,23]. Comparable plasma concentration–time profiles observed after oral and intravenous administration further support this view. Consequently, caffeine consumed in coffee reliably reaches the circulation in physiologically relevant concentrations.

**Figure 1:**
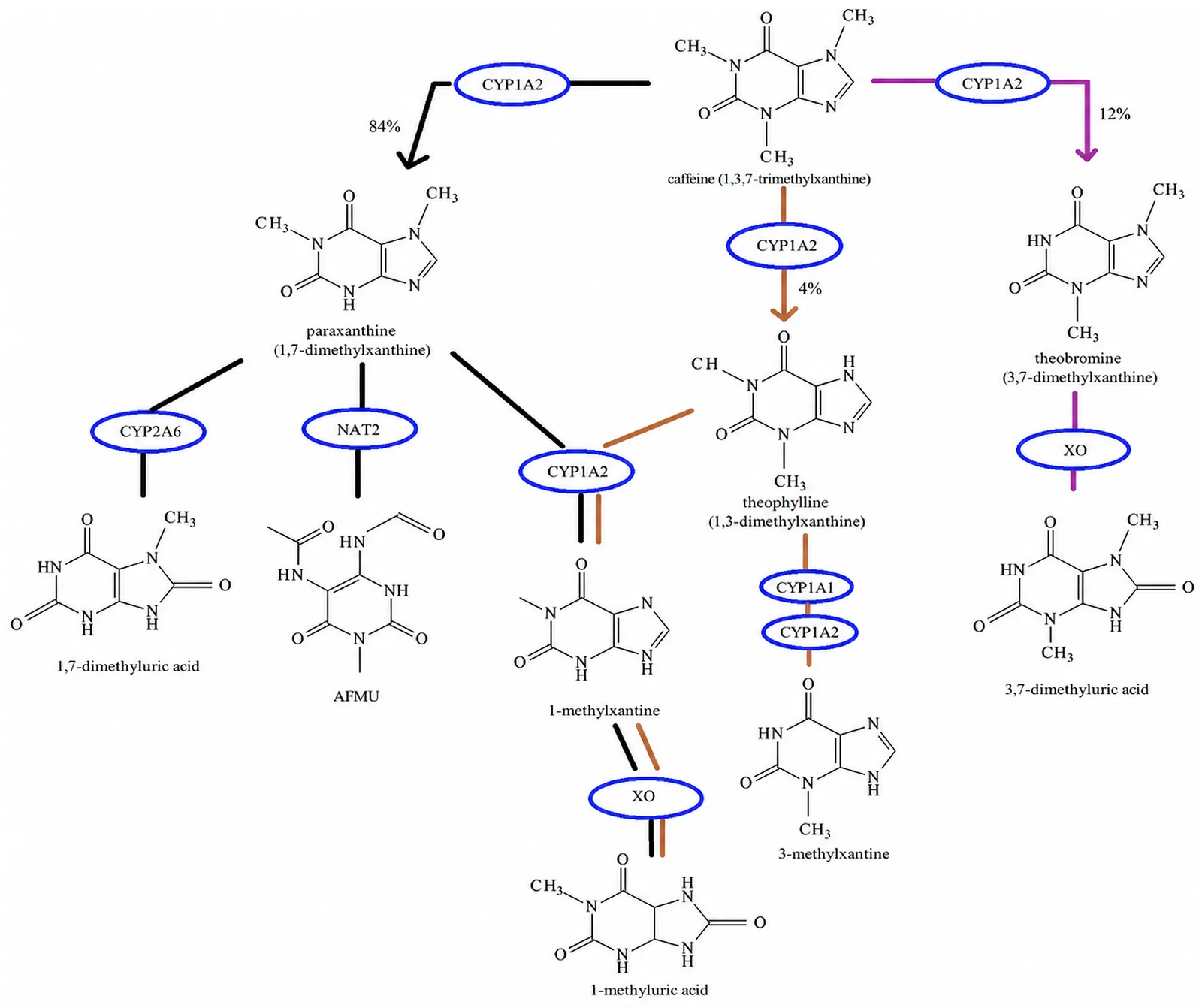
The metabolic pathways of caffeine with CYP1A2. CYP1A2—cytochrome P450 Family 1 Subfamily A Member 2; CYP2A6—cytochrome P450 family 2 subfamily A member; CYP1A1—cytochrome P450 Family 1 Subfamily A Member 1; NAT2—*N*-Acetyltransferase 2; XO—Xanthine oxidase.

The elimination half-life of caffeine is commonly estimated at approximately 4–6 h; however, values ranging from about 1.5 to 10 h have been reported, reflecting pronounced interindividual variability [24,25]. This variability is primarily related to differences in metabolic clearance rather than absorption. The metabolic pathways of caffeine and its primary metabolites are illustrated in Figure 1, highlighting the central role of hepatic cytochrome P450 enzymes in determining the duration of caffeine’s physiological effects.

### 3.2. Genetic Variants of Caffeine Metabolism

Interindividual differences in caffeine metabolism are commonly attributed to genetic variability, particularly polymorphisms within the CYP1A2 gene, which encodes cytochrome P450 1A2—the enzyme responsible for approximately 95% of caffeine metabolism [26,27,28,29]. Individuals carrying the AA genotype typically exhibit higher enzymatic activity and faster caffeine clearance, whereas those with AC or CC genotypes metabolize caffeine more slowly.

CYP1A2-mediated demethylation of caffeine results in the formation of paraxanthine, theobromine and theophylline, accounting for approximately 84%, 12% and 4% of caffeine elimination, respectively [30,31,32], as shown schematically in Figure 1. These metabolites retain biological activity and may contribute to the overall physiological response to caffeine intake [22]. Only a small proportion of caffeine, estimated at 3–5%, is excreted unchanged in the urine [19,33].

In addition to metabolic rate, genetic variation influences sensitivity to caffeine’s central effects. Polymorphisms in the *ADORA2A* gene, encoding the adenosine A2A receptor, have been associated with increased susceptibility to anxiety, nervousness and sleep disturbances following caffeine intake [34]. Importantly, genetic factors should be regarded as moderators rather than determinants of caffeine’s ergogenic effects. Slower metabolism does not necessarily confer a performance advantage and may, in some individuals, increase the likelihood of adverse effects, particularly with repeated or late-day intake.

### 3.3. Influence of Caffeine on Physical Performance

Caffeine intake has been consistently associated with improvements in physical performance, particularly in endurance-based exercise. Evidence from controlled trials and meta-analyses indicates that caffeine can delay the onset of fatigue and reduce perceived exertion during prolonged aerobic activity, with the most reliable effects observed at doses of approximately 3–6 mg/kg body mass [9,10,11,12,13,35,36].

**Figure 2:**
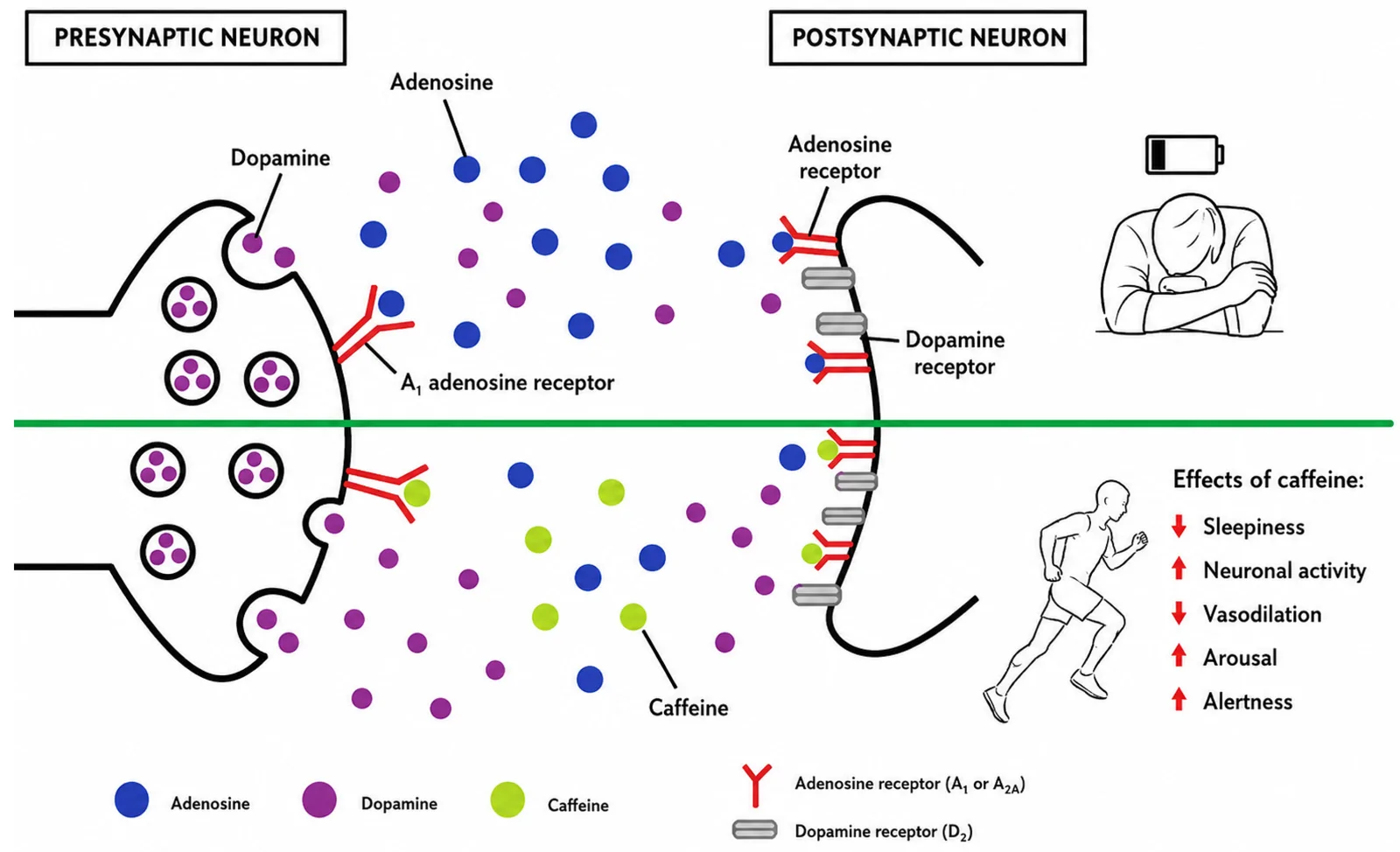
Schematization of adenosine attaching to receptors; this impact is associated with sleepiness and feeling drowsy (Top). (Bottom) Caffeine acts by blocking the transmission of adenosine. Caffeine binds to adenosine receptors; this affects excitatory action and reduces the feeling of drowsiness.

Available evidence indicates that these effects are mainly explained by central nervous system processes, with peripheral mechanisms playing a secondary role. Caffeine acts as an antagonist of adenosine receptors in the central nervous system, thereby attenuating adenosine-mediated inhibitory signaling and sustaining arousal, vigilance and motivation during exercise [35,37,38,39]. This mechanism, illustrated in Figure 2, provides a physiological basis for the commonly reported reduction in perceived effort and improved tolerance to prolonged or demanding exercise.

The timing of caffeine ingestion is another important factor influencing its ergogenic effects and should be considered together with dose. Caffeine is most commonly consumed approximately 60 min before exercise, although the optimal interval may vary depending on the form in which it is ingested [36]. This is particularly relevant when caffeine is consumed as coffee, as the kinetics of caffeine absorption may differ from those observed with other delivery forms [40]. Moreover, in trained males receiving 6 mg/kg caffeine, muscle contraction time was faster 30 min after ingestion than after 60 min, indicating that physiological responses may vary across the pre-exercise period [41]. The response to caffeine may also be influenced by habitual intake and interindividual variability, which should be considered when interpreting performance outcomes [20,21]. Training status may further contribute to variability in caffeine-related performance outcomes, particularly during high-intensity exercise. Some evidence suggests that ergogenic responses may be more apparent in specifically trained individuals; for example, caffeine improved sprint swimming performance in trained but not untrained swimmers. However, findings across exercise modalities are not uniform, and differences in training status and training specificity should therefore be considered when comparing the magnitude and reliability of caffeine effects across studies [40]. In addition, trained athletes generally show lower variability in performance testing, which may facilitate detection of relatively small ergogenic effects [40].

In contrast, the effects of caffeine on strength and power performance are less consistent. While some studies report modest improvements in repeated force production or muscular endurance, effects on maximal strength and explosive power vary substantially between individuals [42,43,44,45,46,47,48,49]. In such contexts, caffeine appears to influence performance primarily by supporting central drive and effort tolerance rather than by directly enhancing maximal contractile capacity.

### 3.4. Muscle Pain and Fatigue Perception

Muscle pain and exercise-related discomfort may contribute to limitations in exercise performance and influence tolerance to strenuous effort [42]. Evidence suggests that caffeine may modulate pain perception associated with exercise and recovery, although the magnitude and timing of this effect vary across studies. In particular, caffeine supplementation has been associated with reduced delayed-onset muscle soreness following exercise, with some evidence suggesting reductions in pain perception during the recovery period rather than a consistent effect during exercise itself [43,44].

**Figure 3:**
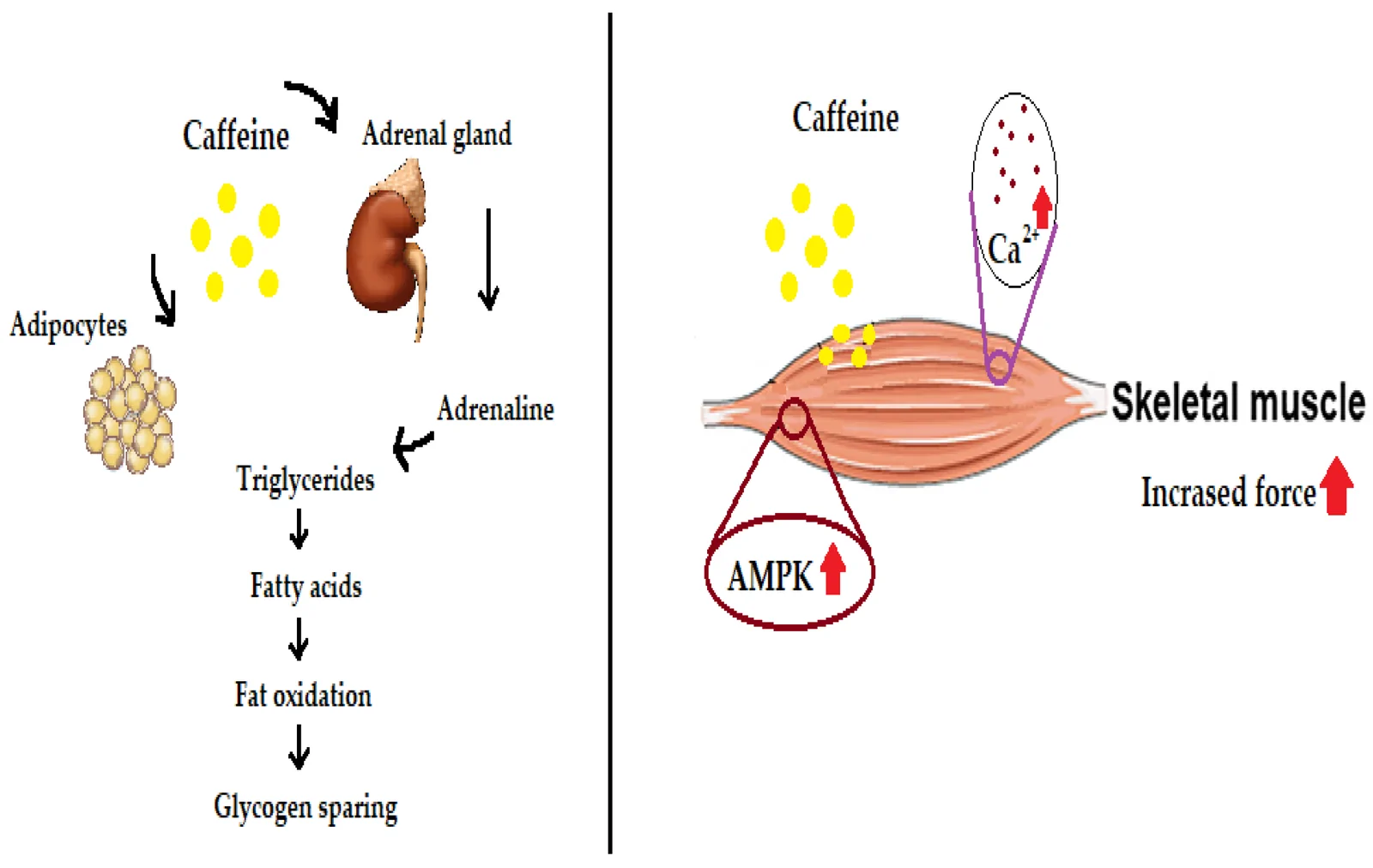
Caffeine increases circulating free fatty acid availability and may alter substrate utilization during prolonged exercise. Evidence for reduced skeletal muscle glycogen utilization is inconsistent, whereas direct evidence regarding hepatic glycogen metabolism remains limited.

These effects are largely mediated by central mechanisms. By modulating neurotransmitter activity and attenuating adenosine signaling, caffeine influences pain perception and central fatigue, making physically demanding exercise more tolerable. The interaction between adenosine receptors and central arousal pathways, depicted in Figure 3, helps explain why caffeine more consistently affects fatigue perception than maximal force production.

At higher doses, caffeine may also exert peripheral effects. Facilitation of calcium release from the sarcoplasmic reticulum has been proposed as one mechanism through which caffeine may attenuate fatigue-related declines in force production during repeated contractions [45,46,47,48,49]. The relationship between caffeine, calcium handling and muscle contractility is schematically illustrated in Figure 3.

### 3.5. Glycogen Metabolism and Recovery

Beyond its acute effects during exercise, caffeine may also influence post-exercise recovery, particularly with respect to muscle glycogen resynthesis. Skeletal muscle and liver glycogen play distinct physiological roles during exercise and recovery. Muscle glycogen serves as the primary fuel source for contracting skeletal muscle, whereas hepatic glycogen is essential for maintaining blood glucose concentrations, especially during prolonged exercise. Although caffeine has long been proposed to affect glycogen metabolism through alterations in substrate utilization, the available evidence differs substantially between these two glycogen pools. Most human studies have examined skeletal muscle glycogen, whereas direct evidence regarding hepatic glycogen utilization or resynthesis following caffeine or coffee ingestion remains scarce.

When co-ingested with carbohydrates during the recovery period, caffeine has been reported to enhance post-exercise skeletal muscle glycogen resynthesis following exhaustive endurance exercise [50,51]. Nevertheless, findings across intervention studies are not entirely consistent and appear to depend on carbohydrate availability, caffeine dose, the extent of glycogen depletion and the duration of recovery [52].

**Figure 4:**
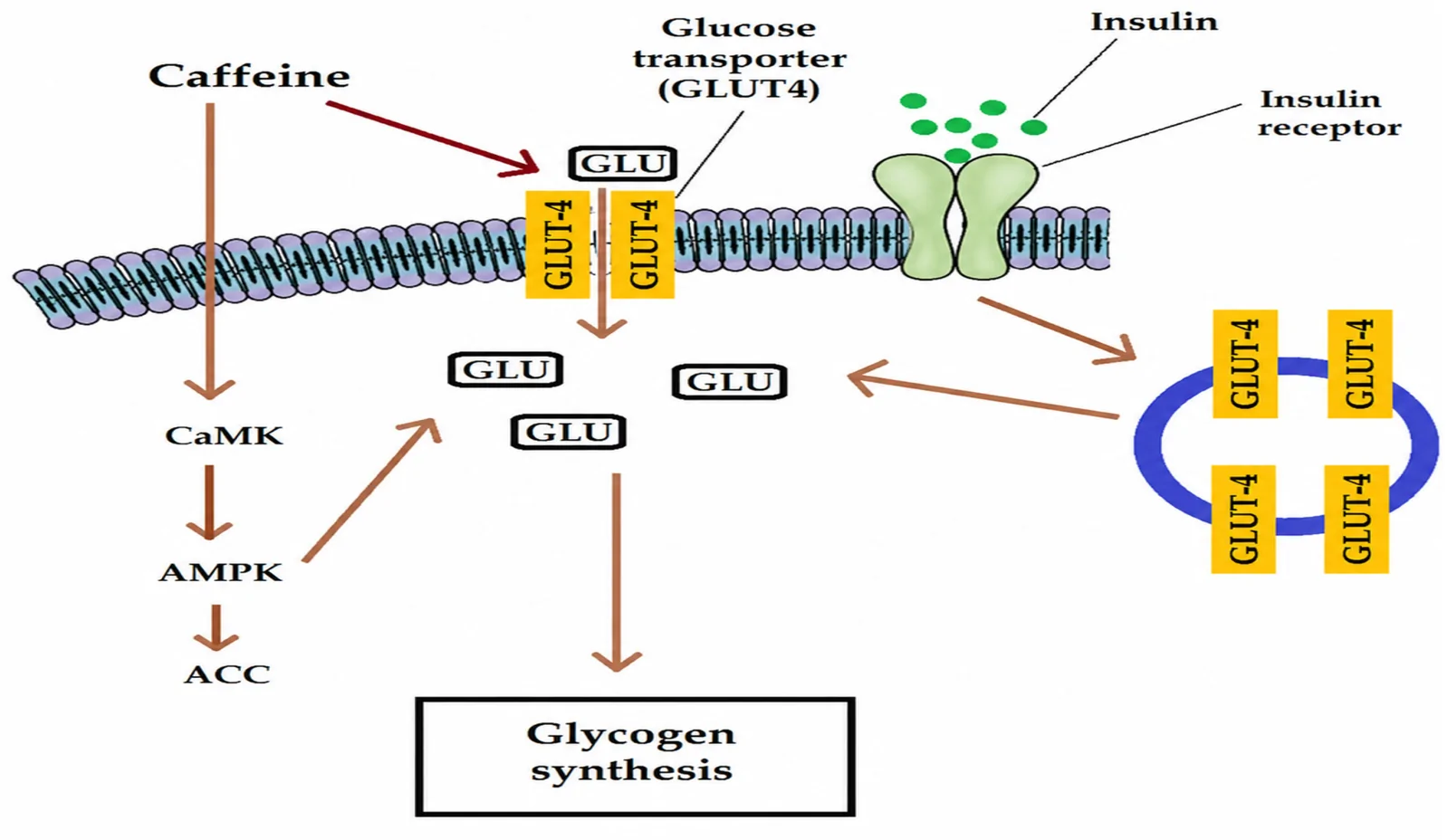
Proposed mechanisms through which caffeine may influence post-exercise skeletal muscle glycogen resynthesis following co-ingestion with carbohydrates. Caffeine may activate the CaMK–AMPK signaling pathway, promoting GLUT4 translocation to the plasma membrane and facilitating glucose uptake. In parallel, insulin stimulates GLUT4 translocation through insulin receptor signaling. Together, these mechanisms may enhance glucose availability for skeletal muscle glycogen resynthesis during recovery. Abbreviations: GLUT4, glucose transporter type 4; GLU, glucose; CaMK, Ca^2+^/calmodulin-dependent protein kinase; AMPK, AMP-activated protein kinase; ACC, acetyl-CoA carboxylase.

The mechanisms responsible for these effects have not been fully established. Increased glucose availability, augmented insulin responses and modulation of glycogen synthase activity have all been proposed to contribute to enhanced glycogen restoration under conditions of low intramuscular glycogen. The interaction between caffeine intake, glucose metabolism and post-exercise glycogen synthesis is summarized in Figure 4. From a practical perspective, these findings appear most relevant to endurance athletes facing short recovery periods between successive training sessions or competitive events.

Early studies investigating the ergogenic effects of caffeine proposed that caffeine might reduce glycogen utilization during prolonged exercise by increasing fatty acid availability and altering substrate utilization. Subsequent controlled human studies, however, have not consistently confirmed reduced skeletal muscle glycogen utilization following caffeine ingestion [53]. Evidence regarding hepatic glycogen metabolism is even more limited because liver glycogen has rarely been assessed directly in exercise studies involving caffeine or coffee. Consequently, current evidence does not support firm conclusions regarding a specific hepatic glycogen-sparing effect.

Evidence for comparable effects following resistance exercise or high-intensity intermittent exercise remains limited. Moreover, any potential benefit of caffeine during recovery should be considered alongside its well-established effects on sleep. When consumed later in the day, caffeine may impair sleep quality and, consequently, recovery and subsequent exercise performance [15,16,17]. Taken together, current evidence suggests that caffeine and coffee may support post-exercise skeletal muscle glycogen recovery under selected conditions, whereas their effects on hepatic glycogen metabolism remain insufficiently understood. Their potential role during recovery should therefore be interpreted in relation to the recovery conditions, particularly carbohydrate availability, timing of intake and the specific metabolic outcome assessed, rather than as a universally applicable strategy.

### 3.6. Caffeine and Circadian Rhythm in the Context of Physical Activity

Circadian rhythm exerts a well-documented influence on physical performance, with exercise capacity in many individuals being lower in the early morning hours and higher later in the day. This diurnal variation appears particularly pronounced for neuromuscular performance variables, such as strength and power, which often peak in the afternoon or early evening [54,55,56]. Despite this physiological pattern, athletes are frequently required to train or compete early in the morning, creating a mismatch between circadian timing and performance demands.

In this context, caffeine is commonly used to counteract circadian-related reductions in alertness and performance during morning exercise. Rather than acting as a regulator of the circadian clock itself, caffeine modulates the functional expression of circadian rhythm by attenuating sleepiness and enhancing central nervous system arousal, particularly under conditions of partial sleep restriction or accumulated fatigue [16,17]. These effects may help reduce the performance gap typically observed between morning and later-day exercise sessions. Pre-exercise caffeine intake may also delay perceived fatigue and reduce ratings of perceived exertion, potentially helping athletes maintain exercise intensity when performance is challenged by circadian factors [29].

The effects of caffeine in this setting may also be influenced by habitual caffeine consumption. However, evidence regarding habituation is not entirely consistent, and habitual caffeine use should therefore be considered as a potential moderator rather than as a simple determinant of the ergogenic response [20,21]. The timing of ingestion is also relevant; however, as discussed in Section 3.3, the optimal pre-exercise interval may depend on the caffeine source and individual response.

Circadian disruption is also relevant in competitive sport settings involving long-distance travel and rapid time-zone transitions. Jet lag is associated with impaired alertness, altered sleep–wake patterns and reduced exercise performance. In such situations, caffeine consumption may temporarily alleviate subjective sleepiness and support daytime alertness during periods of circadian misalignment [16,17]. However, these effects should be regarded as compensatory rather than as evidence that caffeine realigns the underlying circadian system.

At the same time, the use of caffeine to counteract circadian-related fatigue must be balanced against its potential negative effects on sleep. Caffeine intake, particularly later in the day, has been associated with delayed sleep onset, reduced total sleep time and alterations in sleep quality and architecture [15,16]. When repeated evening training sessions are combined with early-morning exercise, residual stimulant effects may therefore interfere with recovery.

Susceptibility to caffeine-related effects varies substantially between individuals and may be influenced by differences in caffeine metabolism and sensitivity [24,28]. Genetic variation in CYP1A2 has also been investigated as a potential moderator of the ergogenic response to caffeine [29]. Accordingly, the timing of caffeine consumption in relation to circadian rhythm and individual metabolic characteristics should be considered when coffee is incorporated into regular training routines.

**Figure 5:**
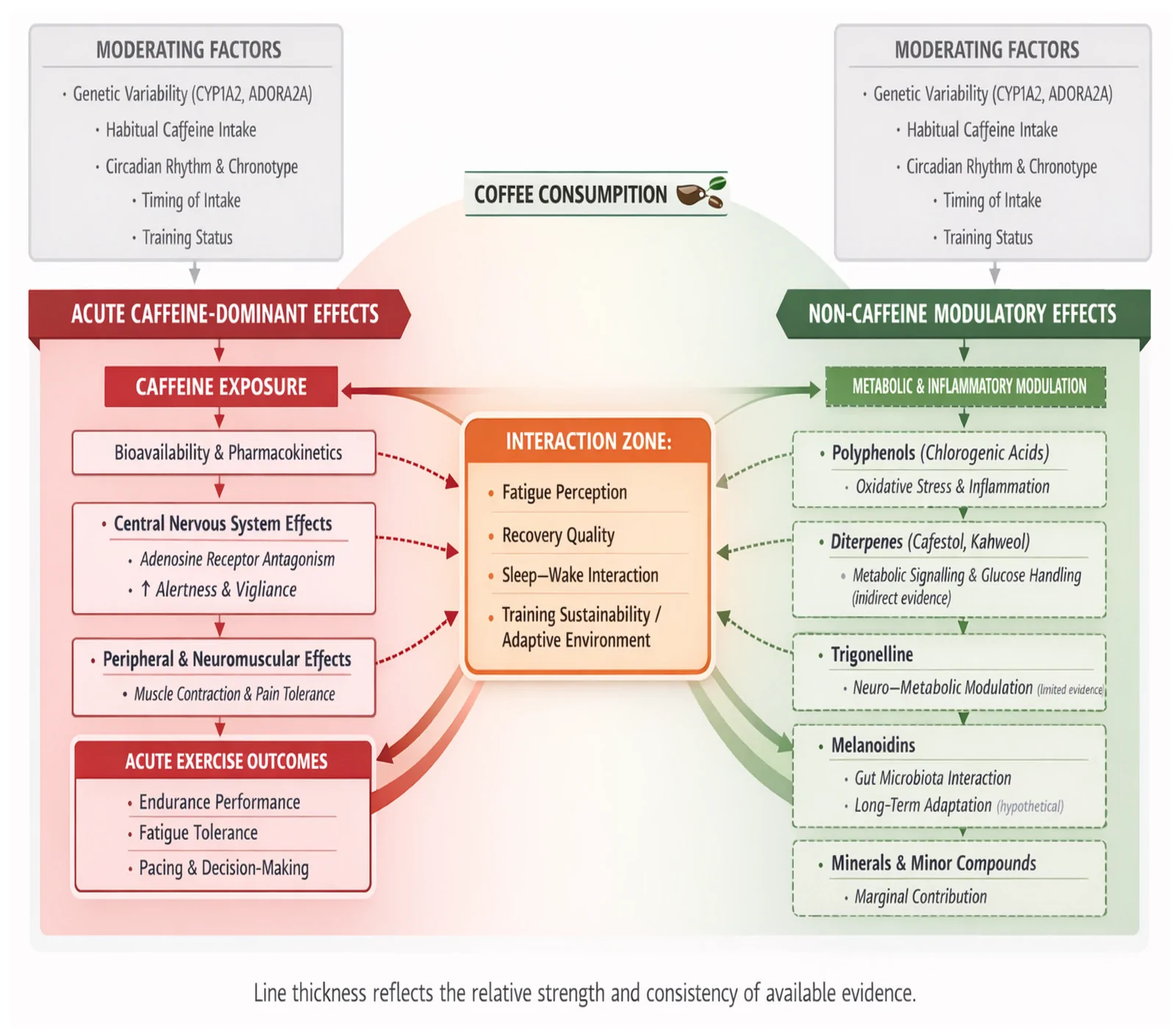
Conceptual framework illustrating pathways through which coffee consumption may relate to physical activity and training-related processes.

To integrate these well-established, caffeine-dominant mechanisms with emerging evidence on other bioactive compounds present in coffee, a conceptual framework distinguishing primary acute effects from secondary modulatory pathways is presented in Figure 5. The figure distinguishes between primary, acute effects predominantly driven by caffeine and secondary, modulatory effects associated with non-caffeine bioactive compounds present in coffee.

The caffeine-dominant pathway encompasses well-established central and peripheral mechanisms, including adenosine receptor antagonism, reduced perception of effort and pain, and acute effects on endurance performance. In contrast, non-caffeine constituents, such as polyphenols, diterpenes, trigonelline, melanoidins and minor minerals, are depicted as modulators of metabolic, inflammatory and recovery-related processes rather than direct, acute ergogenic agents.

An interaction zone highlights shared processes, including fatigue perception, recovery quality and sleep–wake interactions, which may be relevant for the sustainability of training over time. Moderating factors, such as genetic variability, habitual caffeine intake, circadian rhythm, timing of ingestion and training status, are shown to influence both pathways. Line thickness reflects the relative strength and consistency of available evidence supporting each mechanism.

## 4. Evidence for Non-Caffeine Coffee Components in the Context of Physical Activity

Although caffeine represents the most extensively studied bioactive component of coffee in relation to exercise performance, coffee is a chemically complex beverage containing numerous additional compounds with biological activity. Increasing attention has therefore been directed towards the potential contribution of non-caffeine constituents of coffee to exercise-related processes, particularly through their influence on fatigue, recovery and metabolic regulation. Unlike caffeine, evidence for these compounds remains comparatively limited and is derived predominantly from mechanistic, metabolic and recovery-oriented studies rather than from direct exercise–performance outcomes. Accordingly, the following sections focus on the potential physiological relevance of individual coffee constituents while critically considering the current level of evidence supporting their role in exercise and recovery.

### 4.1. Polyphenols

Polyphenols, particularly chlorogenic acids, represent one of the most abundant groups of bioactive compounds in coffee. These compounds exhibit antioxidant and anti-inflammatory properties, which may be relevant in the context of physical activity, given that exercise induces transient oxidative stress and inflammatory responses [57,58,59,60]. While moderate, short-term increases in oxidative stress are widely recognized as an important stimulus for training adaptation during regular training and preparatory phases, excessive or prolonged oxidative burden may impair recovery and contribute to cumulative fatigue.

During competitive periods and repeated high-load events, the primary objective often shifts from maximizing adaptive stimuli to maintaining performance and facilitating recovery between bouts of exercise. In such contexts, strategies aimed at limiting excessive oxidative stress and the associated inflammatory response are commonly employed. Within this framework, coffee consumption in the post-exercise or post-competition period may be considered as a potential supportive element of recovery. Evidence suggests that coffee-derived polyphenols may modulate markers of oxidative stress and inflammation, potentially supporting post-exercise recovery rather than directly enhancing exercise performance [57,58].

In addition, chlorogenic acids have been associated with improved glucose metabolism and insulin sensitivity, which may indirectly influence substrate availability during recovery and subsequent exercise bouts, particularly during periods of intensified training or competition [58,60]. When considered alongside emerging evidence for enhanced post-exercise muscle glycogen resynthesis following coffee consumption combined with carbohydrates, these observations suggest a plausible modulatory role of coffee in recovery-related processes.

Chlorogenic acids may also interact with caffeine-related mechanisms. In vitro evidence suggests that chlorogenic acids may interfere with caffeine-related adenosine receptor activity, potentially attenuating some of its physiological effects. However, the relevance of this interaction in vivo remains uncertain, as the concentrations of chlorogenic acids typically achieved after coffee consumption may be insufficient to substantially modify caffeine action [40].

Importantly, direct evidence demonstrating combined or additive effects of these mechanisms in competitive settings remains limited. Therefore, any potential benefits should be regarded as complementary to established recovery strategies and interpreted in relation to the specific exercise and recovery conditions, rather than as a primary intervention.

### 4.2. Diterpenes

Diterpenes, such as cafestol and kahweol, are lipid-soluble compounds present in coffee, particularly in unfiltered preparations [59]. Experimental evidence indicates that these compounds can influence metabolic signaling pathways, including those involved in glucose handling and lipid metabolism [51]. From the perspective of physical activity, their potential relevance appears to lie primarily in the modulation of metabolic and inflammatory processes rather than in acute enhancement of exercise performance. Observations related to glucose regulation and glycogen-associated pathways may therefore be considered of mechanistic interest, although their translation to exercise-specific outcomes remains uncertain.

Available evidence concerning diterpenes is largely derived from cellular and metabolic studies, with limited data directly examining exercise performance or recovery in physically active populations. Consequently, any contribution of cafestol and kahweol to physical activity should be interpreted as indirect. Their potential effects may be more relevant during recovery or repeated training exposure, where metabolic regulation plays a greater role, than during single bouts of exercise.

### 4.3. Trigonelline

Trigonelline is an alkaloid naturally present in green coffee beans, with concentrations decreasing substantially during the roasting process. Experimental studies have attributed neuroprotective and metabolic properties to trigonelline, including potential effects on glucose metabolism and neuronal function [57]. In the context of physical activity, these properties suggest a possible modulatory role in fatigue perception or metabolic regulation. However, the available evidence is derived predominantly from studies using green coffee or isolated trigonelline, whereas beverages prepared from green coffee remain relatively uncommon in habitual consumption.

Direct evidence linking trigonelline intake from roasted coffee beverages to exercise performance or recovery outcomes is scarce. Consequently, trigonelline should be regarded as a minor coffee constituent with plausible biological activity, but without clearly established relevance to exercise-specific endpoints. Any potential contribution to physical activity is therefore likely to be indirect, with its relevance depending on the specific metabolic or recovery-related outcome considered.

### 4.4. Melanoidins, Gut Microbiota and the Gut–Muscle Axis

Melanoidins are high-molecular-weight compounds formed during the roasting of coffee through Maillard reactions. These compounds exhibit antioxidant properties and have been shown to interact with the gut microbiota, with available evidence suggesting a predominantly modulatory influence on inflammatory and metabolic processes [50,51]. Experimental and observational studies indicate that melanoidins may promote favorable changes in gut microbial composition and activity, which could be relevant in the context of physical activity. Regulation of gut microbiota may be particularly important for endurance athletes, as prolonged or repeated exercise bouts are known to reduce splanchnic blood flow, leading to transient intestinal ischemia, increased intestinal permeability and translocation of luminal components into the circulation. These exercise-induced gastrointestinal disturbances may contribute to systemic inflammation, gastrointestinal symptoms and impaired recovery.

Within this framework, melanoidins have been proposed as potential modulators of gut microbial composition and function, which may indirectly attenuate exercise-related inflammatory and metabolic perturbations. Such interactions are sometimes discussed within the broader concept of the gut–muscle axis, referring to bidirectional links between gut-derived signals and skeletal muscle metabolism. However, evidence supporting these interactions in relation to coffee-derived melanoidins remains preliminary and largely indirect.

Recent human studies have provided further insight into the interactions between coffee, caffeine and the gut microbiota, suggesting that these relationships are more complex than previously recognized. Habitual coffee consumption has been associated with reproducible changes in gut microbial composition and metabolic activity. Interestingly, similar microbiota responses were observed following both caffeinated and decaffeinated coffee, suggesting that these effects cannot be attributed solely to caffeine and are likely mediated by multiple coffee constituents [61]. In parallel, short-term caffeine supplementation has also been shown to modify gut microbial diversity and composition in trained athletes, although it remains unclear whether these microbiota alterations contribute causally to the observed improvements in exercise performance or simply accompany the physiological effects of caffeine [62]. Together, these observations indicate that interactions between coffee and the gut microbiome are likely mediated by multiple bioactive compounds rather than caffeine alone. Accordingly, the relevance of melanoidins in the context of physical activity should still be regarded as exploratory. While their influence on gut microbiota, systemic inflammation and metabolic regulation may plausibly support training tolerance and recovery, direct evidence linking melanoidin intake to exercise performance or adaptation is currently lacking. Their role should therefore be interpreted as a potential long-term modulatory mechanism rather than an acute or primary ergogenic effect. Among the various non-caffeine constituents of coffee, melanoidins remain one of the most plausible candidates for mediating microbiota-related effects, although their specific contribution has yet to be established.

### 4.5. Minerals and Minor Compounds

Coffee contains small amounts of minerals, most notably potassium (approximately 90–120 mg per cup), along with magnesium (5–10 mg per cup) and niacin (0.5–1.5 mg per cup), as well as various minor bioactive compounds [63]. While these constituents contribute to the overall nutritional profile of coffee, their quantities generally represent only a small fraction of daily requirements and are insufficient to exert meaningful effects on physical performance or recovery in isolation [63]. Accordingly, their relevance for exercise-related outcomes is likely limited to their contribution within the broader dietary context rather than as independent functional factors.

### 4.6. Compounds Influencing Fatigue and Pain Perception

In addition to individual compounds, coffee should be considered as a composite matrix in which multiple constituents may interact to influence central fatigue and pain perception. While caffeine remains the primary driver of these effects, non-caffeine components may contribute indirectly through modulation of inflammatory mediators or neurotransmitter-related pathways [50,57].

Importantly, these potential effects are subtle and unlikely to translate into acute ergogenic benefits. Instead, they may influence subjective experiences of effort and discomfort, particularly during repeated or prolonged training exposure. At present, evidence supporting such interactions remains indirect and warrants further investigation.

Taken together, available evidence indicates that non-caffeine bioactive compounds present in coffee may influence physical activity primarily through modulatory effects on metabolic, inflammatory and recovery-related processes, rather than through direct enhancement of exercise performance. Compared with caffeine, the supporting evidence is less consistent and often indirect. These compounds are therefore best viewed as contributors to the broader physiological context in which training and recovery occur, rather than as independent ergogenic agents.

This difference from the evidence available for caffeine reflects the current state of the literature, where evidence for individual non-caffeine coffee constituents remains substantially less developed and is derived predominantly from mechanistic rather than exercise-specific studies.

## 5. Discussion, Limitations and Practical Implications

The present review synthesizes current evidence on the role of coffee consumption in the context of physical activity, with particular emphasis on caffeine-related mechanisms and the potential modulatory contribution of non-caffeine bioactive compounds. Taken together, the available literature supports the view that the acute effects of coffee on exercise performance are predominantly driven by caffeine, whereas other coffee constituents may influence fatigue, recovery and metabolic processes indirectly, with the magnitude and relevance of these effects varying according to the compound, exercise conditions and outcome assessed [55].

Evidence regarding caffeine consistently indicates beneficial effects on endurance performance, primarily through central mechanisms such as reduced perception of effort and fatigue, enhanced alertness and improved tolerance to sustained exercise. These effects appear robust across a wide range of endurance-based activities, while findings related to strength and power outcomes remain more variable [42,43,44,45,46,47,48,49]. Importantly, interindividual differences related to genetic variability, habitual intake, circadian rhythm and timing of ingestion substantially modulate both the magnitude and reliability of observed responses. As such, caffeine should not be regarded as a universally effective ergogenic aid, but rather as a tool whose utility depends on individual and contextual factors.

In contrast, evidence concerning non-caffeine bioactive compounds present in coffee is less consistent and largely indirect. Available mechanistic and metabolic studies suggest that these compounds may influence processes such as oxidative regulation, glucose and lipid metabolism, and gut microbiota, although the relevance of these effects to exercise-specific recovery remains uncertain [57,58,59]. Direct evidence linking individual non-caffeine coffee constituents to measurable improvements in exercise performance remains limited. Their contribution is therefore best interpreted as modulatory, shaping the physiological environment in which training and recovery occur rather than directly enhancing acute performance outcomes. This distinction is central to avoiding overinterpretation of mechanistic findings and is reflected in the conceptual framework presented in Figure 5.

Several limitations should be acknowledged. First, this review is narrative in nature and does not include formal quality assessment or meta-analytic synthesis, which limits the ability to quantify effect sizes or compare outcomes across studies. Second, the heterogeneity of study designs, exercise protocols and coffee preparations complicates direct comparisons and generalization of findings. Third, much of the evidence relating to non-caffeine compounds is derived from metabolic or mechanistic studies rather than from exercise-specific interventions, highlighting the need for well-controlled trials examining whole-coffee consumption in physically active populations.

From a practical perspective, coffee may be considered a functional component of sports nutrition when used deliberately and with awareness of individual responses. Caffeine-containing coffee consumed prior to exercise may support endurance performance and reduce perceived effort, particularly in situations involving high physiological or cognitive demands. However, timing of intake is critical, as late-day consumption may impair sleep quality and compromise recovery. Potential benefits associated with non-caffeine compounds are likely to manifest over longer time frames and should not be expected to produce immediate performance enhancement.

Future research should aim to clarify the role of whole-coffee consumption in exercise settings, with particular attention to interindividual variability, sex-specific responses, chronotype and repeated use across different phases of training. Studies integrating performance outcomes with markers of recovery and sleep will be especially valuable in refining practical recommendations.

## 6. Conclusions

Coffee consumption may support physical activity and training when used deliberately and with consideration of individual and contextual factors. Available evidence indicates that the acute effects of coffee on exercise performance are predominantly driven by caffeine, particularly through central mechanisms that reduce perceived effort and fatigue and support endurance performance. These effects are most consistent in endurance-based exercise, while responses related to strength and power remain more variable.

Responses to coffee consumption differ between individuals and may be influenced by genetic variability, habitual caffeine intake, timing of ingestion, circadian rhythm, training status and interactions with sleep and recovery. These factors may affect both the magnitude of the ergogenic response and the tolerability of caffeine-containing coffee. Therefore, its use in sports nutrition should be individualized according to the athlete’s characteristics, training demands and timing of consumption rather than assumed to provide a uniform performance benefit.

Non-caffeine bioactive compounds present in coffee may contribute indirectly to exercise-related processes by modulating metabolic, inflammatory and recovery-related pathways. However, evidence supporting direct performance effects of these compounds remains limited, and their role should be interpreted as modulatory rather than ergogenic.

## Data Availability

No new data were created or analyzed in this study. Data sharing is not applicable to this article.
